# Co-producing public involvement training with members of the public and research organisations in the East Midlands: creating, delivering and evaluating the lay assessor training programme

**DOI:** 10.1186/s40900-017-0056-0

**Published:** 2017-04-05

**Authors:** Adele Horobin, George Brown, Fred Higton, Stevie Vanhegan, Andrew Wragg, Paula Wray, Dawn-Marie Walker

**Affiliations:** 1 National Institute for Health Research (NIHR) Nottingham Hearing Biomedical Research Centre, Ropewalk House, 113 The Ropewalk, Nottingham, NG1 5DU UK; 2 Nottingham University Hospitals NHS Trust, Queen’s Medical Centre, Derby Road, Nottingham, NG7 2UH UK; 3 Lay representative, Nottingham, UK; 4 National Institute for Health Research (NIHR) Nottingham Biomedical Research Centre, Queen’s Medical Centre, E Floor, West Block, Derby Road, Nottingham, NG7 2UH UK; 5 National Institute for Health Research (NIHR) Collaboration for Leadership in Applied Health Research and Care East Midlands, Diabetes Research Centre, Leicester General Hospital, Leicester, LE5 4PW UK; 6 National Institute for Health Research (NIHR) INVOLVE Central Co-ordinating Centre, Alpha House, University of Southampton Science Park, Chilworth, Southampton, SO16 7NS UK; 7 Health Sciences, 45/2021, Highfield Campus, University of Southampton, Southampton, SO17 1BJ UK

**Keywords:** Lay assessor, Public, Reviewing, Training, Cross-organisational, Regional, Co-production

## Abstract

**Plain english summary:**

Members of the public share their views with researchers to improve health and social care research. Lay assessing is one way of doing this. This is where people, drawing upon personal and general life experience, comment on material, such as grant applications and patient information, to highlight strengths and weaknesses and to suggest improvements. This paper reports on setting up a training programme for lay assessors.

Meetings were held between interested public and staff from research organisations. People discussed what lay assessing is, why they want to do it, skills and support needed and if training was wanted. They were invited to form a group to develop the training together. Training was delivered in the East Midlands. People who attended gave their thoughts about it by completing questionnaires and joining a feedback event.

The group developed the structure of the training programme together and it oversaw the development of the training content by individual members. People who attended training reported feeling more confident about lay assessing. This was particularly so for those who had not done lay assessing before. They indicated how valuable it was to talk with others at the training. Our findings support the National Institute for Health Research recommendations for improving learning and development for public involvement in research.

This project has created a solid base for local research organisations to work together in public involvement training. Lay assessor training is now part of a wider programme of shared resources called the Sharebank.

**Abstract:**

**Background**

Involving members of the public in research can improve its quality and incorporate the needs and views of patients. One method for doing this is lay assessing, where members of the public are consulted to improve research materials. This paper documents the establishment of a pilot training programme for lay assessors. It describes a way of working that embodies a regional, cross-organisational approach to co-producing training with members of the public.

**Methods**

Open meetings, led by AH, were held for existing and aspiring lay assessors to define lay assessing, motivations for doing it, skills required, associated learning and development needs, and to gauge interest for training. Those who attended meetings, including members of the public and staff, were invited to form a working group to co-produce the training programme. Training was delivered in modules at two centres in the East Midlands and evaluated through participant feedback at the end of each module and at an evaluation event. Feedback was through a mix of Likert scale scoring, open text and verbal responses.

**Results**

Discussions from the open meetings informed the development of the training by the working group. Led by AH, the working group, as a whole, co-produced the structure and format of the training and oversaw training content development by individuals within the group. Training was well-received by participants. Feedback through Likert scoring (*n* = 14) indicated higher feelings of confidence in knowledge of relevant subject matter and in fulfilling the lay assessor role, particularly amongst those who had not done lay assessing before. Opportunities that the training afforded for interaction between participants – sharing of varied experiences and knowledge – and a ‘learn by doing’ approach was of particular value, as indicated by 10 responses to open-ended questions.

**Conclusions**

This project has created a solid foundation for collaboration between research organisations in the East Midlands in devising and delivering training in public involvement together. Our evaluation provides evidence in support of National Institute for Health Research (NIHR) recommendations on principles for learning and development for public involvement in research.

**Electronic supplementary material:**

The online version of this article (doi:10.1186/s40900-017-0056-0) contains supplementary material, which is available to authorized users.

## Background

The National Institute for Health Research (NIHR) supports the opinion that “Research that reflects the needs and views of the public is more likely to produce results that can be used to improve health and social care.” [1]. It is now widely recognised that public involvement should be an essential part of producing research which meets this key criterion. That is, research carried out ‘with’ or ‘by’ members of the public rather than ‘to’, ‘about’ or ‘for’ them [2]. The NIHR, one of the leading funders of health research in the UK, requires that researchers must involve the public in a way which can have a genuine impact on improving research. The NIHR Research Design Service makes clear that “Demonstrating PPI [public involvement] and the continued involvement of patients and members of the public is a very important part of developing a successful grant application and is often a marker of quality research” [3] pp.3. The demonstrable input of patients and public throughout the research cycle, before and after funding has been acquired, is therefore clearly required.

A recognised method for public involvement consists of consulting with patients and the public for their views on various research materials. Views can be drawn from personal experience of taking part in research or from experiencing a health condition or social care issue that is the subject of the research. These can yield deep insights which can only be obtained from having the relevant lived experience. Views can also be shared on more general issues around clarity of language and layout and on the most appropriate ways that the public can be involved. The materials can then be improved in response to the feedback obtained. Referred to as lay assessing^1^, this method can help ensure that:

- Grant applications and protocols include study plans that are relevant to patient need and are appropriately designed for participants.
- Patient information leaflets and consent forms are clearly laid out, written in plain English and highlight all key points of importance to participants so that they can make a genuinely informed decision about taking part in research.
- Recruitment posters and other publicity material are suitably designed to attract the interest of potential participants.
- Dissemination (communication) material, sharing the outcomes of research, are suitably designed and targeted to participants, clinicians, policy makers and the wider public.

For lay assessing to be meaningful and have impact, it is essential that lay assessors and researchers alike realise the breadth of contribution that lay assessors can make to improving research. This is to draw the very most from people’s experiences as a patient, carer, or as a previous participant in research. In some cases, this may require giving information, guidance and support, in the form of training, to enable high quality assessments from lay assessors and subsequent appreciation and considered responses from researchers.

Training initiatives for public involvement are few, with most restricted to individual organisations or integrated within individual research projects [4]. The current siloed working, where public involvement training initiatives are not readily visible or co-ordinated as part of an organisation’s strategy, presents barriers to shared learning across organisations, sharing of resources between organisations and accessibility to people (researchers and public) who are new to public involvement. It presents a particular challenge for offering support in public involvement activities associated with the pre-funding stages of the research cycle, before any grant-awarded funding has been received for supporting public involvement in a study. These issues have been recognised and in response, the NIHR Clinical Research Network is supporting the role-out of the Building Research Partnerships programme [5]. This one day training programme offers an introduction to public involvement and organisations within regions are encouraged to work together in delivering it. The NIHR Research Design Service now offers small research development bursaries for supporting public involvement activities in developing grant applications [6].

Siloed working in public involvement training also brings into question whether the public involvement training available could satisfy the demand for public contributors who have enough familiarity of public involvement in research to fully contribute to the research effort [7]. It has been suggested that public contributors are being drawn from a relatively small pool [7, 8] and it is generally acknowledged locally that this is the case. This is supported by findings that “Rather than training contributors, researchers favoured using their networks and others’ recommendations to select individuals who already possessed attributes perceived as important for the role” pp11 [9]. Taking this approach may restrict the number and diversity of people that can contribute to research. In turn, this may restrict the variety of different perspectives which informs the research. This could undermine the relevancy and suitability of research for different communities.

The reported reluctance to offer training to public contributors, for fear of professionalising or taming the lay perspective [9–11], is also a perspective that needs further examination. A meeting that was held in January 2013 for existing and aspiring lay assessors of Nottingham University Hospitals NHS Trust Research and Innovation Department (NUH R&I) indicated a willingness locally amongst public contributors for supported learning. Evidence suggests that training can help public contributors feel more confident and empowered to take a critical approach [11]. The case for training to give public contributors “sufficient background to be confident and effective in their involvement role” has also been eloquently described pp186 [12]. Public contributors at the meeting also expressed the need for guidance on what makes for good quality assessments of material, and opportunities to learn from one another. This supports INVOLVE’s suggestion that public involvement training should encompass a learner-centred approach, with participants taking an active role in their learning [13]. The literature also indicates that training should be co-produced with public contributors, starting from the needs of the learners, focusing on real research problems, being specific to roles in public involvement and offering opportunities for interaction and sharing of participants’ experiences [7–9, 11, 13].

This paper describes a way of working together that embodies a coordinated and cooperative, cross-organisational and regional approach to public involvement training for lay assessing. The intended outcomes were three-fold – i) to create a pilot training programme for lay assessors, thus plugging the gap in resources for public involvement training at the ‘pre-funding’ stage; ii) to deliver the programme; and iii) evaluate the public involvement training delivered. This paper reports on how these outcomes were achieved.

## Methods

### Creating the pilot training programme for lay assessors

Our approach to developing the pilot training programme was one built on collaboration and inclusivity. Collaboration, in that a working group of members of the public and members of staff from across research organisations was assembled to develop the programme together, as an example of co-production with the public. Inclusive, in that working group membership was open to any public and local/regional organisations interested in taking part. Guided by the working group, the training was designed to be learner-centred, promoting active learning, or learning by doing.

As described in the Introduction, a meeting that was held in January 2013 for existing and aspiring lay assessors of NUH R&I, (prompted by the cessation of lay assessor training at the Trust) indicated a willingness amongst local public contributors for supported learning on lay assessing. This prompted work to develop the lay assessor training programme.

Work started with two further, open meetings, led by AH and involving the following organisations: NIHR Nottingham Hearing Biomedical Research Unit, NIHR Nottingham Digestive Diseases Biomedical Research Unit, NIHR East Midlands Research Design Service and NUH R&I. These were to provide the foundations for developing a new training programme and to build a sense of ownership of the project, generating the interest to create a more formal working group. Meetings were held in the format of small group discussions (5-6 per group, facilitated by a public involvement professional), followed by a plenary. They were each attended by over 20 people, approximately 15 of whom were members of the public involved in research. Around half of these had also attended the initial meeting in January 2013.

At the first of these two meetings (August 2013), discussions were guided to obtain a shared view going forwards around:

- What lay assessing is
- Why people want to do lay assessing
- The skills required and associated learning and development needs.

At the second of these two meetings (October 2013), discussions were guided around how we can instil the principles for learning and development recommended by the INVOLVE led cross-NIHR working group [13]. These principles are:

- That ongoing support should be provided
- Support is accessible to all
- Learning is appropriate and relevant to the task
- Individual readiness to learn is acknowledged and builds on existing knowledge and abilities (pp.16).

Notes taken by facilitators for each discussion group formed the basis for the analysis of each meeting. An inductive approach (developing ideas from what was reported) was taken [14], with the aim of reporting respondents’ views, grouped under themes that AH perceived to be present in the notes.

Participants of these two meetings appeared engaged and enthused to see a new training programme developed. They were therefore invited to create a working group to develop the pilot training programme for lay assessors. For inclusion, people were accepted with a range of public involvement experience, from none to five or more years in a variety of contexts. The working group was chaired by AH, who also recorded minutes for each meeting. Minutes from the previous meeting were reviewed and agreed at each subsequent meeting. Public members of the working group were offered honorary payments and expenses costs.

The working group had 17 members, comprising 12 members of the public and 5 professional public involvement staff representatives from organisations in the region. Members represented a range of experiences of illness and disability. They also included two retired education academics and one person with experience of designing and delivering training courses. Organisations represented were the NIHR Nottingham Hearing Biomedical Research Unit, NIHR Nottingham Digestive Diseases Biomedical Research Unit, NIHR Collaboration for Leadership in Applied Health Research and Care East Midlands, NIHR East Midlands Research Design Service and NUH R&I: The group first met in November 2013. Numbers attending each meeting ranged from 8 to 13 members. Six meetings were held in total.

The working group was tasked to:

- Agree on which of the themes identified by the previous open meetings were relevant to developing the training programme and could feasibly be adopted in the initial pilot.
- Co-produce the structure and format of the pilot programme including defining topics of content and how these should be delivered.
- Co-produce the training content.
- Agree evaluation plans and associated materials.

Willing members of the working group were also tasked to deliver the training programme, whether that be through acting as lead trainers, facilitators and/or identifying and booking suitable venues. Trainers did not undergo any training themselves and instead, drew upon their own experiences - professionally or personally - from being involved in research.

### Delivering the pilot training programme for lay assessors

Members of the public were recruited to the pilot lay assessor training programme by invitation from public involvement professionals who were members of, or known to, the working group. The public members recruited were already involved in research projects known to the public involvement professionals or had recently expressed an interest in getting involved but had various levels of experience in public involvement. Participants were encouraged to attend the entire programme but this was not mandatory. Refreshments during training and travel and/or carer expenses were provided. No fees were charged for attendance.

Public involvement professionals within the working group and others in the region facilitated discussions during training sessions. Participants sat in small groups around tables and held discussions within their groups on planned activities. Opportunities were given for participants to report points raised during discussions to everyone else taking part. Time was given for refreshment breaks during the training.

Prior to delivering the programme, AH obtained £3000 funding from the East Midlands Academic Health Science Network to cover non-staff costs associated with launching the programme. This included honorary payments and expenses for working group public members who contributed to the development and delivery of the programme, resource materials printing, refreshments and expenses payments to participants. Honorary payments were optional and in line with NIHR recommendations. Expenses included local travel costs and carer attendance costs.

### Evaluating the pilot training programme for lay assessors

The programme was evaluated using the Kirkpatrick’s training evaluation model [15] shown in Table 1, modified to replace Results (which focuses on the wider effect of the training) with Future Development (as this represents the next step for this pilot programme). Data were collected for the evaluation by asking participants to complete pre-course questionnaires (delivered by email shortly before people attended the programme), post-course questionnaires (delivered by email 1 month following completed attendance) and feedback forms (delivered at the end of each module). Responses were captured using a mix of Likert scale ratings (range of scores = 1 to 6, with 1 indicating a negative/unsatisfied response and 6 indicating a positive/satisfied response) and free text options.

**Table 1:** Evaluation plan, based on modified version of Kirkpatrick’s training evaluation model

Focus of evaluation	Questions	Criteria	How measured
Reaction to the course	How well was the training material delivered?	Perceived quality of presentation Level of detail provided Pace of delivery Amount of audience interaction/participation	Feedback form completed by trainees at end of each training session
Learning	Did the course modules fulfill the learning goals?	Level of confidence trainees had about their knowledge of topics relevant to each module Level of confidence trainees had on how prepared they felt for lay assessing	Feedback form completed by trainees at end of each training session
Influence	Did attending the course have an impact on trainees’ confidence in their knowledge relating to lay assessing?	Change in level of confidence trainees had about their knowledge of topics relevant to lay assessing after attending the course	Comparison of pre- and post-course questionnaires completed by trainees
	Were there any lasting impacts from the course on trainees?	New public involvement roles/activities adopted by trainees	Self-reports from trainees
Future development	What are trainees’ main learning and development needs following completion of the training course?	Ongoing support requested	Evaluation discussion event with working group and trainees

In the pre- and post-course questionnaires, respondents were asked to indicate whether they had done lay assessing before and were then asked to rate their feelings of confidence of knowledge on:

- The research cycle
- Research funding sources
- What information goes into a grant application form
- The role of the lay assessor in improving a grant application
- The basics of research methods in health research
- The basics of ethical issues in health research
- The work of Research Ethics Committees
- Patient confidentiality
- The meaning and importance of ‘informed consent’
- What information goes into a participant information sheet
- The role of the lay assessor in improving a participant information sheet
- The purpose of other study-related documents (e.g. protocol, promotional flyers, progress updates)
- Roles in patient and public involvement other than lay assessing.

Data from named respondents who completed both pre- and post-course questionnaires were compared.

In the feedback forms, respondents were asked to give their views on the quality of presentation, the level of detail provided, the pace of delivery and the amount of audience interaction and/or participation. They were also asked to rate their confidence in their knowledge of areas covered in the module and how prepared they felt to conduct aspects of lay assessing relating to the module in question. These areas of knowledge mapped on to those areas included in the pre- and post-course questionnaires.

In addition, all working group members and programme participants were invited to attend an evaluation event in Nottingham in February, 2015. The meeting was held in the format of small group discussions (5-6 per group, facilitated by a public involvement professional), guided by pre-set questions posed by the lead author to prompt participants to reflect on their experience and discuss learning and development needs going forwards. Notes taken by facilitators for each discussion group at each meeting formed the basis for analysis. An inductive approach [14] was taken, with the aim of reporting the views of the respondents, grouped under themes that AH perceived to be present in the notes.

Guidance from the Health Research Authority advised that this work did not require NHS Research Ethics Committee approval as it would not be classified as research within the NHS. According to the Health Research Authority, research requiring ethical review includes participants randomised to different groups, involves changing treatment or patient care from accepted standards or offers findings that are generalizable to a broader patient population [16]. Rather this work is the establishment and evaluation of a training service, co-produced with public involvement. We therefore sought and obtained a favourable ethical opinion from the University of Nottingham Ethics Committee [Additional file 1]. Quotes given in feedback from participants were anonymised and permission was obtained from participants before their quotes were reported.

## Results

### Creating the pilot training programme for lay assessors

The key purpose of the training was identified as providing enough background knowledge, through examples and sharing of participants’ own experiences and knowledge, to support participants in developing the confidence to formulate and express their own challenging questions when lay assessing a piece of work. Themes that were identified from discussions of the two open meetings (held in August and October 2013) represented the starting point for developing the lay assessor training programme. The working group considered which of the themes were relevant and which could be feasibly adopted in the initial pilot.

As shown in Table 2, discussions around the theme of ‘topics of learning’ informed the structuring of the training programme into 3 modules:

**Table 2:** Themes identified from open meetings, which informed training development by the working group

Theme	Integration into training
**Topics of learning**	
Basic understanding of research	Content created on research methods and ethics – formed first training module.
Understand the context: • Research process that lay assessing fits into • Role in relation to other public involvement roles	• Content included to show and explain research cycle and public involvement roles that fit into points on the cycle. Game included to help participants understand stages that must be navigated before research can start. • Two modules created which pivot around stages in research – lay assessing pre-funding and lay assessing post-funding.
What research organisations exist and key contacts	List of local research organisations and key contacts included in training.
Confidentiality	Highlighted as part of training on ethics in research.
Intellectual property	Not included.
Diplomacy	Public involvement professional facilitators available to offer ‘insider’ viewpoints.
**Approach to learning**	
Learn by doing	• Activities built in to training, using real life research examples. • Opportunity to conduct lay assessment of a grant application.
Guidance rather than diktat	Emphasis placed on participants coming up with their own answers, through discussion.
Social support: • Sharing experiences and ideas • Mentoring	• Group activities included, to prompt discussion between participants. • Establishing a mentoring scheme was deemed beyond the scope of the pilot programme, due to resource limitations.
**Impact of learning**	
Increased confidence to question and challenge	• Emphasis placed on discussions between participants. • How participation affected perceived confidence in role as lay assessor was included in evaluation of training.
Opportunity for progression in public involvement	• List of local research organisations and key contacts included in training. • Information on national resources (People in Research, INVOLVE) included in training. • Information on involving the public in service improvement (Patient Participation Groups in primary care) included in training. • Public involvement roles other than lay assessing highlighted in training. • Reports from participants on their public involvement activities following training was included in evaluation of training.
**Accessibility of training**	
Public involvement in development of training	Working group, including public members, co-produced the training.
Different access routes: • Face-to-face – group training or one-to-one if preferred. • Training material available online • Paper versions of online material	Group face-to-face training offered only, with paper-based resources to take away. Other options were deemed beyond the scope of the pilot programme, due to resource limitations.
Advertise widely	Decision taken to advertise internally to members of the public, who have already had contact with public involvement professional members of the group, to ensure that demand could be managed and to maximise use of existing networks.
**Flexibility of training**	
Training optional	Public invited to attend but not conditional to doing public involvement work.
Training according to people’s preferences and needs	• Participants not obliged to attend all three training modules. • Participants could attend at one or both training centres.

Bold type indicates overarching themes identified

1. Research Methods and Ethics, aimed to build a basic understanding of research, research methods, ethical issues and lay assessment in relation to the role of research ethics committees;
2. Lay Assessing Pre-funding, aimed to build understanding of the process that leads to commencing research, lay assessing the grant application form and other roles in public involvement.
3. Lay Assessing Post-funding, aimed to build understanding of lay assessing patient information materials, study protocols and a refresher on the importance of informed consent (covered in the first module).

Each module also included information on contact details of public involvement staff that participants could approach for involvement opportunities.

Discussions on the theme of ‘approach to learning’, indicated that an active learning approach was preferred, where participants taken an active role in their own and others’ learning. The working group fully supported this preference. Thus, opportunities for group activities and participant discussions were built into the training. Real-life examples of research material, produced locally and elsewhere representing a broad spectrum of healthcare research, were used for participants to examine and practice elements of lay assessing. Also included was a ‘board game’ for participants to complete. Akin to ‘Snakes and Ladders’ the board game guided participants through the stages that need to be completed before a research study can start. While the working group supported the idea of facilitating formal mentoring, it was decided that this could not be achieved as part of the initial pilot, due to restrictions on staff time.

Discussions on the ‘impact of learning’ theme influenced evaluation planning for the training programme. The working group agreed that participants’ levels of confidence in their knowledge of research and their role as lay assessors should be key indicators of the impact of training. Indicators of progression in public involvement experience were included in evaluation plans.

Discussions around the theme of ‘accessibility of training’ were considered. While the working group was supportive of creating varied access routes to training (face-to-face and online), it was decided this could not be achieved as part of the initial pilot, due to restrictions on staff time. The working group agreed to focus on developing group face-to-face training only. The group also agreed to advertise training only to existing contacts, in order to manage demand.

On the theme of ‘flexibility of training’, the working group agreed that there should be a flexible approach. Training was always optional and participants were not obliged to attend all three modules of training.

Module 1 training content was developed by a public member (GB) of the working group, while modules 2 and 3 were developed by a public involvement professional (AH) of the working group. Content was supplemented with real-life research case examples provided by public involvement professional members of the group.

Discussions from the first open meeting (August 2013) around what lay assessing is, why people want to do it and the skills required informed the development of a lay assessor role profile. This was developed by a separate, smaller working group of public and public involvement professionals (not described here). The role profile was included in the training content and is therefore included in this paper as an additional file [see Additional file 2]. The role profile distinguished lay assessing based on personal experience of a health condition relevant to the research and that based on a general knowledge, with assessors encouraged to consider both approaches.

### Delivering the pilot training programme for lay assessors

The pilot lay assessor training programme was delivered face-to-face at two centres in the East Midlands – Nottingham (September 2014) and Leicester (January 2015). At each centre, the programme was delivered over three half-days (each half-day covering one module and delivered one week apart). In Nottingham, GB and AH delivered the training content that they had each developed (GB delivered module 1 and AH delivered modules 2 and 3). In Leicester, AH delivered modules 1, 2 and 3, as GB was unavailable.

The programme was delivered to 30 participants in total, of whom 15 attended all three modules. The Nottingham centre was attended by 11 participants and 4 public involvement professionals (including AH) who facilitated discussions. The Leicester centre was attended by 21 participants and 5 public involvement professionals (including AH) who facilitated discussions. Two of the participants had attended both Nottingham and Leicester in order to complete all three modules. Participants who didn’t complete all three modules were not able to attend on the days the programme was run.

Following feedback on the delivery of module 1 at Nottingham, AH made a number of changes to the resources for the subsequent delivery of module 1 at Leicester.

### Evaluating the pilot training programme for lay assessors

Of the 30 participants who attended the course, 22 (73%) completed the pre-course questionnaire and 17 (57%) completed the post-course questionnaire. Of these, 14 (47%) had completed both the pre- and post-course questionnaires. Only responses from those who had completed both the pre- and post-course questionnaires were included in the analysis. Two of these 14 respondents had omitted recording a Likert score feedback on one of thirteen listed options where respondents were asked to rate their feelings of confidence of knowledge on each (on the pre-course questionnaire for one respondent and the post-course questionnaire for the other). Any scoring on this option from these respondents was therefore removed from the analysis. One non-responder for the post-course questionnaire did not attend any of the training, despite completing a pre-course questionnaire. There were no noticeable traits amongst other non-responders. Response rate on module feedback form completion and return ranged from 75% (6 out of 8 people) to 100% per module delivery.

#### Reaction to the course

End of module feedback showed that the training was welcomed and well received. In overview, ratings were high (Table 3) and written comments were positive. Of particular prominence were comments which indicated participants particularly valued the level of audience interaction and that this aided learning:

**Table 3:** Respondents’ scores on feedback forms where 1 = least satisfied and 6 = most satisfied

	Module 1 (*n* = 21) Research methods and ethics (IQR)	Module 2 (*n* = 21) Lay assessing pre-funding (IQR)	Module 3 (*n* = 22) Lay assessing post-funding (IQR)
Quality of presentation	6 (4.5 to 6)	5 (5 to 6)	6 (5 to 6)
Detail provided	6 (5 to 6)	5 (5 to 6)	6 (5 to 6)
Pace of delivery	6 (5 to 6)	5 (5 to 6)	6 (5 to 6)
Audience interaction	6 (5 to 6)	6 (5 to 6)	6 (5 to 6)

Median Likert score, with the interquartile range (IQR) shown in brackets

“A well-structured informative workshop which was rightly predominantly interactive and therefore a powerful learning and bonding event.” P 27

The important contribution of interaction to learning was also highlighted by a participant who was very open about their learning difficulties and felt that the course offered the right amount of interaction (P 29). Other participants also indicated that the opportunity to interact and discuss with others was valued (P 2, 17, 32, 35).

Further comments indicated that the audience valued the mix of experiences in the room and this included an acknowledgement of the role of public involvement professionals who took part in discussions (P 3, 6, 31).

A suggestion was also made to increase the opportunity for interaction still further by moving tables during group exercises to work with other people too, giving added weight to its value (P 32).

#### Learning

The ratings shown in Table 4 indicate a very positive response to the three modules of the course in terms of learning. Respondents valued the knowledge that they had gained (P 12, 16, 27, 31, 34) and related its usefulness to their role as a lay assessor (P 32, 34).

**Table 4:** Respondents’ scores on feedback forms where 1 = least satisfied and 6 = most satisfied

	Median Likert score (IQR)
Module 1 (*n* = 19)	
**Confidence in knowledge**	
The basics of research methods in health research	5 (4 to 6)
The basics of ethical issues in health research	5 (4 to 5)
The work of Research Ethics Committees	5 (4 to 6)
**Confidence in practising/examining**	
Ethical dilemmas in study proposals	5 (4 to 6)
Potential weaknesses in research methods	5 (4 to 5)
Module 2 (*n* = 20)	
**Confidence in knowledge**	
The process to leading up to commencing research	5 (4 to 5.75)
What information goes into a grant application form	5 (4 to 5)
The role of the lay assessor in improving a grant application	5.5 (4.25 to 6)
Roles in public involvement other than lay assessing	5 (4.25 to 6)
** Confidence in practising/examining**	
Undertaking lay assessing of grant applications	5 (4.25 to 6)
Module 3 (*n* = 21)	
**Confidence in knowledge**	
Where lay assessing post-funding fits in to process leading up to commencing research	5 (5 to 6)
The range of materials that lay assessors may come across at the post-funding stage	5 (5 to 6)
The meaning and importance of ‘informed consent’ (*n* = 20)	6 (5 to 6)
The role of the lay assessor in improving a participant information sheet	6 (5 to 6)
** Confidence in practising/examining**	
Undertaking lay assessing at the post-funding stage	5 (5 to 6)

Median Likert score with interquartile range (IQR) shown in brackets. Bold type indicates overarching themes

The snakes and ladders board game guiding participants through the process of establishing a research project and real life examples, such as the Northwick Park^2^ study patient information sheet, were amongst elements of the course that were specified as useful for supporting learning (P 16, 19):

“Snakes and Ladders game was a hit – [public representatives] were really thinking about the different things that go in research, before funding.” (Personal communication from public involvement professional facilitator).

Games have been used in public involvement activities before and have shown to be effective tools for exploring issues and aiding learning [17, 18].

#### Influence

The influence (or impact) of the training was evaluated through analysing responses from the pre-and post-course questionnaires to see if the course had increased participants’ sense of confidence about their knowledge of research and how they can contribute. Email and phone contact were also sought as follow up to find out if attending the course had helped participants in getting more involved in research.

**Fig. 1:**
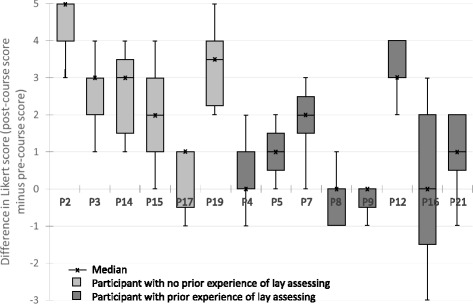
Title: Comparison between respondents’ pre- and post-course questionnaire scores. Legend: Data expressed as the median difference between pre- and post-score, with interquartile range (*box*) and minimum and maximum shown (*whiskers*)

Figure 1 shows that the majority of trainees scored more highly in confidence one month following training. The increase was higher amongst those trainees who had not undertaken any lay assessing before starting the course. The effect on confidence was not so marked amongst those with prior experience of lay assessing. This is not necessarily to say that this group did not feel more informed but the emphasis of the course was not around delivering ‘facts’. It was more around empowering trainees to question and probe researchers on their research, thus realising the breadth of contribution that lay assessors can make. To this end, the course had a greater impact on those who were naïve to lay assessing.

Personal communications with the lead author revealed that as a direct result of attending the training course, one person was recruited to a local service user-led research network (P 19). Another participant successfully acquired a paid public involvement position with a research institute, having found the position through Partners in Research website. This resource had been highlighted on the course. The participant was extremely appreciative of how doors were opened because they could state that they had been trained in public involvement: “Exciting to go through your course then go out there and use it” (P 2). Another participant was inspired enough to apply to do a PhD using a methodology which treats the community being studied and the participants as equal partners in the study (P 6). A participant who was already involved in a research project on a long term health condition when joining the course stated that the training had helped with their public involvement role:

“It was really interesting and informative talking to PPI [public involvement] members involved with research into other Long Term Conditions, particularly those dealing with the Mental Health aspects of LTC [long term conditions]” P 12

#### Reflections on feedback and revisions to the programme

For the first delivery of module 1, the pace of delivery and audience interaction was rated highly, but the reaction to the quality of the presentation and detail provided was mixed, with nearly half of respondents giving lower ratings. Written responses on the feedback forms reflected this response, with concerns being raised about the quality of the presentation, including the graphics and colours used (P 3), the lack of recommended pre-course reading (P 2) and insufficient details of the research process to bolster understanding of what a lay assessor is involved with (P 8).

Consequently, the presentation for module 1 was modified: the layout and colour scheme of the presentation was made consistent with the other modules. Additional handouts and slides were created to help better explain research methods. In addition, a short list of recommended reading was sent to the second group of participants before they started module 1.

The modifications appeared to make an improvement in feedback scores to module 1. Associated written comments were positive. Respondents indicated their enjoyment of the session and that the presenter and information presented was clear and thus aided learning (P 12, 16, 19, 31).

#### Future development

Twenty people attended the evaluation event to discuss future development of the course. This included 16 course participants (of whom 7 were members of the working group) and 4 staff representatives (public involvement professionals, of whom 3 were members of the working group). Responses centred on being given the opportunity to put learning into practice – real-life lay assessment opportunities and practice examples. A way of accessing resources outside of the course was also sought, including information available online, discussion forums and a central resource for finding opportunities to get involved. In addition, participants expressed the desire to see more input from researchers and have opportunities to interact with researchers. They also indicated that they would like to keep in contact with other lay assessors to share experiences, sustain interest and aid continued learning.

## Discussion

This project documented the value that can be created from co-ordinating a cross-organisational regional approach to public involvement training, co-produced with members of the public. The initial open meetings with the public and staff were vital in confirming the demand for training. They also provided the opportunity for people to get to know one another and to set the tone of cooperation and inclusion. The meetings allowed people to make an informed decision about getting involved further and thus stimulated the interest and commitment for creating a working group focused on developing the training. The meetings also provided information which guided the working group in developing the training. However, the working group was free to implement or reject suggestions that were made, based on what was practical to deliver.

It is interesting to note that the working group, as a whole, had the greatest input into deciding the modular structure and format of the lay assessor training programme, the length of training sessions that participants would be prepared to engage in and to whom the training programme should be advertised. Extensive discussions were essential for this. Also, the key purpose of the training, which the working group agreed should be in providing enough background knowledge to support participants in developing the confidence to scrutinise, independently corresponded with that put forward by Staley [12]. The working group was also pivotal to ensuring that an active learning approach was taken, through having oversight of training content that was being developed. Creation of the training content itself evolved to be reliant on particular members of the working group as it became clear that this was not going to be developed through group discussion. However, having professional representation from a number of research organisations on the working group was particularly helpful in sourcing real-life examples of research that could be incorporated into the training. Through tapping in to each organisation’s network of contacts, this cooperative, cross-organisational approach was central to recruiting enough participants towards a ‘critical mass’ which could generate fruitful discussions and sharing of a wide range of experiences.

Indeed, the evaluation of the course highlights the importance of the social dimension to learning. Opportunities to share experiences and information with others appeared to be a key aspect to participants’ favourable response to the course. In addition, comments directly linked social interaction with learning. Our findings support those of Lockey and colleagues [8] who found that a “key aspect of successful training was exchange and sharing between people, both trainers and participants” (pp.1) and the more recent work of Gibson and colleagues [11] which indicated that a learning environment promoting discussions between lay trainees consolidated learning. The mix of experiences within the room was also valued and this concurs with Lockey and colleagues [8] and Gibson and colleagues [11]. It also highlights the valuable role of public involvement professionals in enriching the learning experience and indicates potential for further widening of the mix of participants. There may be value in researchers and public learning with and alongside one another. Indeed, feedback at the evaluation event included the request for researchers to be more involved in the training. In support of this assumption, Gibson and colleagues [11] reported a positive response from lay participants to the presence of academic and clinical professionals in training workshops.

Participants responded well to a course that was focussed on specific tasks and real examples, associated with a well-defined role. Dudley and colleagues’ [9] work, exploring the opinions of researchers and public contributors towards public involvement training, highlighted a preference for training which clarifies role expectations. Their findings also advocated for researchers and public to learn alongside one another in order to develop good relationships and mutual understanding of roles.

It is interesting to note that following training, participants reported high levels of confidence in their knowledge of subject matter relating to lay assessing and in facing the task of doing lay assessing. Parkes and colleagues [7], Dudley and colleagues [9] and Gibson and colleagues [11] highlighted a role for training to help public contributors confidently challenge researchers in order to overcome the perceived power imbalance between researcher and public contributor. Amongst our course participants, an increase in feelings of confidence in fulfilling the lay assessor role was more pronounced amongst those who had not done lay assessing before. This may indicate the particularly valuable role that training can offer to help widen the pool of willing public contributors, who, as a result of training, may be more willing to put themselves forward and get involved.

One important lesson we learned from delivering the course was the need to continually reflect upon what is being delivered and react accordingly. The mixed response that we had to the first delivery of module 1 (Nottingham), prompted us to revise the module materials and presentations. This resulted in a much more favourable response for the second delivery. Evaluation should be an ongoing activity, rather than something to be left until the end [15]. Another lesson we learned was that in planning the training, it would have been preferable to include provisions for ongoing support. From the evaluation event, it was clear that course participants were eager to have the opportunity to (1) put their learning into practice as soon as possible, with real projects and/or practice examples, (2) continue access to educational resources and (3) have further interactions (both peers and researchers). While public involvement professionals who contributed to the training invited participants to get involved in projects, it was not possible to deliver ongoing support to all participants. A move away from an *ad hoc* approach towards continuity of public involvement beyond single initiatives was not achieved in this respect. The short term financial support for this project, and an unsuccessful request for follow-on funding, contributed to this situation and highlights the ongoing difficulty in putting public involvement initiatives on a sustainable footing. However, it was encouraging that a number of course participants reported becoming significantly more involved in research as a result of gaining confidence and new contacts from attending the training. Continued regional working may improve continuity of public involvement through improvements in sharing resources and communicating opportunities.

One criticism that could be made of our work is that the systematic follow up of all the course participants could have been extended beyond one month after training. It would be useful to see how many of the original participants were still involved in research, and whether they still draw upon the benefits they gained from the course. It must also be stated that in any situation where people self-select to attend (or not) a training programme, there is the potential for selection bias. This is where those who attend the training may not necessarily represent everyone who can have a valuable input in research. While our evaluation feedback is positive, caution must be applied to recognise the possibility of non-response bias (where non-responders may have lower satisfaction with the course than those who responded) and that the number of reporting participants is relatively small. It would have been ideal if the training had been evaluated by individuals who had not previously been involved in developing and delivering the training. This is to ensure complete impartiality in the interpretation of the feedback and minimise the risk of responders modifying their feedback out of politeness or gratitude. However, resources were not available to do this.

Nevertheless, we believe that our report gives considerable insight into how to establish the co-production of public involvement training and how to ensure that the training delivered is engaging, relevant and influential. It also demonstrates the advantages of taking a regional approach to training as opposed to training individuals involved in one project or in one organisation. This approach allowed for larger class sizes and thus greater scope for interaction and learning from the mix of health and research experiences present.

Our course evaluation also provides evidence in support of NIHR’s recommendations on principles for learning and development for public involvement in research [13] (pp.16). These four principles are:

i. Provides ongoing support in three key areas – administrative, research and personal support
ii. Is accessible to all
iii. Is appropriate and relevant to the task
iv. Acknowledges individual readiness to learn and builds on existing knowledge and abilities.

We strived to embed the principle of accessibility including offering expense payments to course participants, allowing flexibility on attendance, providing information in plain English (with provision made for vision impairment as requested), providing breaks and refreshments and sourcing accessible venues. We had built in appropriateness and relevance through harnessing active participation to build on participants’ existing skills, experience and prior knowledge, providing tasks based on real examples so participants ‘learn by doing’ and using feedback to improve our course. We had also acknowledged participants’ readiness to learn through involving the public in delivering training, encouraging debate and questioning, managing mixed ability groups, sharing explicit learning objectives and providing a variety of different approaches to learning (including verbal and visual presentations, group work and paper-based). These measures were all well-received and ways to develop and extend still further in these aspects were indicated in the feedback. However, we had not been able to provide the ongoing support that had been requested, including mentoring, online forums and face-to-face opportunities for further discussions. There was also no systematic way to ensure that all participants subsequently had real opportunities for getting involved in research. A lack of sustainable funding hampered our ability to develop these aspects, begging the question as to whether all four principles should be embedded from the start of any training initiative, rather than relying on staged development. This is perhaps where cross-organisational involvement in developing training can be advantageous through sharing of opportunities and maintaining a ‘critical mass’ of interest for follow-up events and online forums.

## Conclusions

As a result of this project, establishing coordination across organisations and co-production of public involvement training has created a solid foundation for further collaborative activities. Indeed, this has spawned the establishment of the public involvement training ‘Sharebank’ to deliver training and support for public involvement [19]. Based on organisations sharing resources on a reciprocal basis, without the need to pay for training, this initiative will help public involvement training and support gain a more sustainable footing. It has already helped to evolve the lay assessor training programme, which has been adopted into the Sharebank initiative. In response to feedback and knowledge of new course lead facilitators, the programme has been modified by the NIHR East Midlands Collaboration for Leadership in Applied Health Research and Care and the NIHR East Midlands Research Design Service for delivery to targeted audiences. However, the core of the course has been maintained and has been delivered in a collaborative spirit.

## Additional files

Additional file 1: EV16012017 Dr Adele Horobin PPI Training programme evaluation Research Ethics Committee letter. (PDF 68 kb)

Additional file 2: Lay assessors in research. Role summary for a lay assessor. (PDF 604 kb)

## Abbreviations

INVOLVE: National advisory group that supports active patient and public involvement in NHS, public health and social care research
NHS: National Health Service
NIHR: National Institute for Health Research
PPI: Patient and public involvement

## Footnotes

1 Lay assessing is the term used in this training programme. INVOLVE, the national advisory body on public involvement in research, refers to the term public reviewing. Lay reviewing is another alternative.

2 Northwick Park study refers to a clinical study conducted in 2006 at Northwick Park hospital. The study tested a monoclonal antibody called TGN1412, developed by TeGenero Immuno Therapeutics, in human participants. It caused very serious adverse reactions in the participants, leading to systemic organ failure.
