# Supplementary material for: Co-producing public involvement training with members of the public and research organisations in the East Midlands: creating, delivering and evaluating the lay assessor training programme
Source: Res Involv Engagem. 2017 Apr 5;3:7. doi: 10.1186/s40900-017-0056-0 (PMC5611661; doi:10.1186/s40900-017-0056-0)
Supplement: Supplementary file 2 — Lay assessors in research. Role summary for a lay assessor. (PDF 604 kb) [file 40900_2017_56_MOESM2_ESM.pdf]

Lay Assessors in Research

The purpose of this guide is to provide a clear idea of what a lay assessor is and what the role involves. Useful attributes to assist in your role are listed. Contact details are given.

1. Who are lay assessors?

Lay assessors are members of the public, such as you. You do not require any formal qualifications and are not expected to be an expert in any specific field. You contribute your own experience and independent view through reading and commenting on proposed research projects. Lay assessors may also be known as 'lay reviewers'. The 'lay assessor' is one of many roles for the public in research.

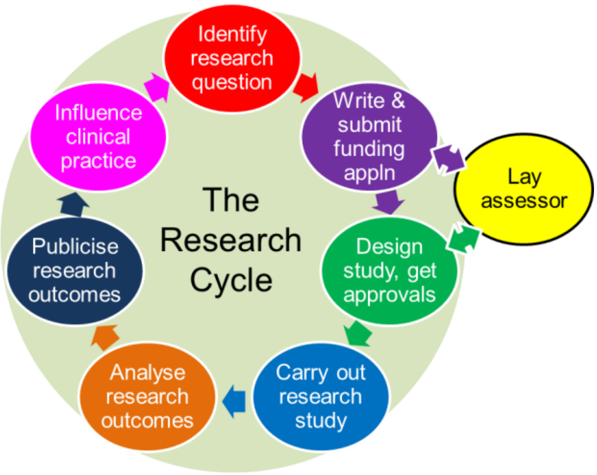

2. What is the role?

Before a research project can start, researchers must:

- Get funding for their project
- Get ethical approval for their project. This includes approval of all information sheets given to participants.

An application for funding includes a broad outline of the research and how the research will be done. It also includes who will do the research, how much it will cost and when it will be completed. Participant information must provide a clear summary of the project. It must also describe what is asked of the participant.

"You may review proposed applications for research funding"

As a lay assessor, you may be asked to read and comment on the whole funding application, or parts of it. For example, you may be asked to feedback on the way patients will be recruited into the study.

"You may review participant information sheets and consent forms"

You may be asked to comment on how clearly information sheets are written. You may also comment on whether research participants are being given all the information that they need.

You will review material using your own experiences to offer fresh views on the suitability of the study proposed and the information provided.

"You are not expected to have or use technical or expert knowledge to judge the details of the research methods"

As a lay assessor you will work on your own but will benefit from a support framework around you.

"Your own viewpoint is important"

You and other assessors will be asked to give individual viewpoints, which the researcher will consider when making revisions to the material assessed.

Guide co-produced by Dr Adele Horobin (NIHR Nottingham Hearing Biomedical Research Unit) with members of the public and colleagues from Nottingham University Hospitals NHS Trust. October, 2013.

3. Why do people become lay assessors?

There are many reasons why you might decide to become a lay assessor. Some are suggested below:

- To help improve research and learn more about it.
- To ‘give something back’ for care or treatment that you, or someone you know has received.
- To act on personal experience or the experience of family or friends.
- To speak for others who can’t speak for themselves and to ensure that the patient experience is not ignored.
- To learn more about a health condition.
- To increase confidence and develop new skills.
- To contribute without having to travel or attend team meetings. This may be of particular attraction if you have work or other commitments or have mobility issues.

4. What activities make up lay assessment?

Before completing a lay assessment, you may be contacted by a member of the research team or a Patient and Public Involvement (PPI) Facilitator.

You will then receive a copy of the research project application or participant information. You will be asked to read and comment on it.

The researcher may ask you specific questions or you may be asked to submit your comments on a standard form. You will complete the form based on your own understanding of the material provided.

The researcher or PPI Facilitator will acknowledge your contribution and provide feedback.

5. What support can you expect as a lay assessor?

Your PPI Facilitator will give you guidance and any additional support that you might need. You may be given the opportunity to meet other lay assessors if you so wish.

Once invited to assess a research project, you are free to accept or decline the invitation, without having to give a reason. You are free to withdraw from the assessment process at any stage, if you wish.

All reasonable expenses will be paid. You will be informed whether or not you may receive payment, above expenses, for your work.

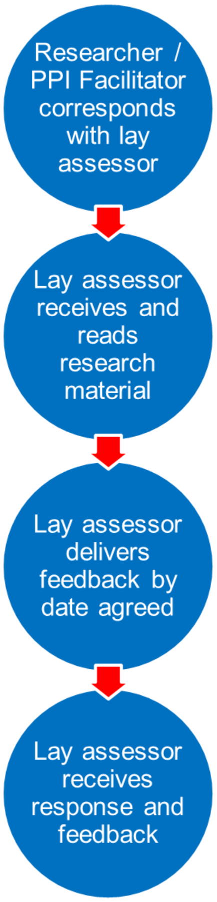

Process of lay assessment

Guide co-produced by Dr Adele Horobin (NIHR Nottingham Hearing Biomedical Research Unit) with members of the public and colleagues from Nottingham University Hospitals NHS Trust. October, 2013.

6. What qualities are required of lay assessors?

Top 10 key attributes for lay assessors:

**1**	**An interest in research to improve healthcare.**
**2**	**The willingness to spend time getting to understand the information given.**
**3**	**Being able to respond clearly in writing.**
**4**	**The confidence to ask questions and challenge assumptions.**
**5**	**Understanding that your opinion is important to researchers.**
**6**	**The ability to challenge the use of jargon and acronyms.**
**7**	**Keeping an open mind.**
**8**	**The ability to see things from a general point of view.**
**9**	**Being able to put yourself in someone else's shoes.**
**10**	**Happy to draw upon your own experiences or your knowledge of others' experiences.**

Personal experience
You can work on research projects about health conditions you have experienced. This may be as a patient, carer, relative, friend or colleague. You can also work on health conditions that you have not come across. Here, you would use your own general knowledge.

7. How should lay assessors and researchers conduct themselves?

Lay assessors and researchers are expected to:

- Be well organised, so as to able to meet the deadline agreed.
- Treat people with empathy, courtesy and respect and value others' opinions.
- Maintain the confidentiality of all the information they receive.

8. Other roles for the public in research

Do you think lay assessing is for you? Not sure? There are many other ways in which you can get involved with research. Members of the public may contribute in all stages of the research process.

You could be part of the research team. You could join a focus group and contribute to answering a question for the research team.

You could join an organisation's patient advisory group. You could help publicise research outcomes and advocate for putting outcomes into clinical practice.

You can also work for funding authorities, providing a lay perspective to submitted funding applications.

Please speak to your Patient and Public Involvement Facilitator for more details:

Dr Adele Horobin, Patient and Public Involvement & Engagement Manager

NIHR Nottingham Hearing Biomedical Research Unit. Tel. 0115 823 2614
Email. adele.horobin@nottingham.ac.uk
Website. www.hearing.nihr.ac.uk

Guide co-produced by Dr Adele Horobin (NIHR Nottingham Hearing Biomedical Research Unit) with members of the public and colleagues from Nottingham University Hospitals NHS Trust. October, 2013.

9. How you may influence research

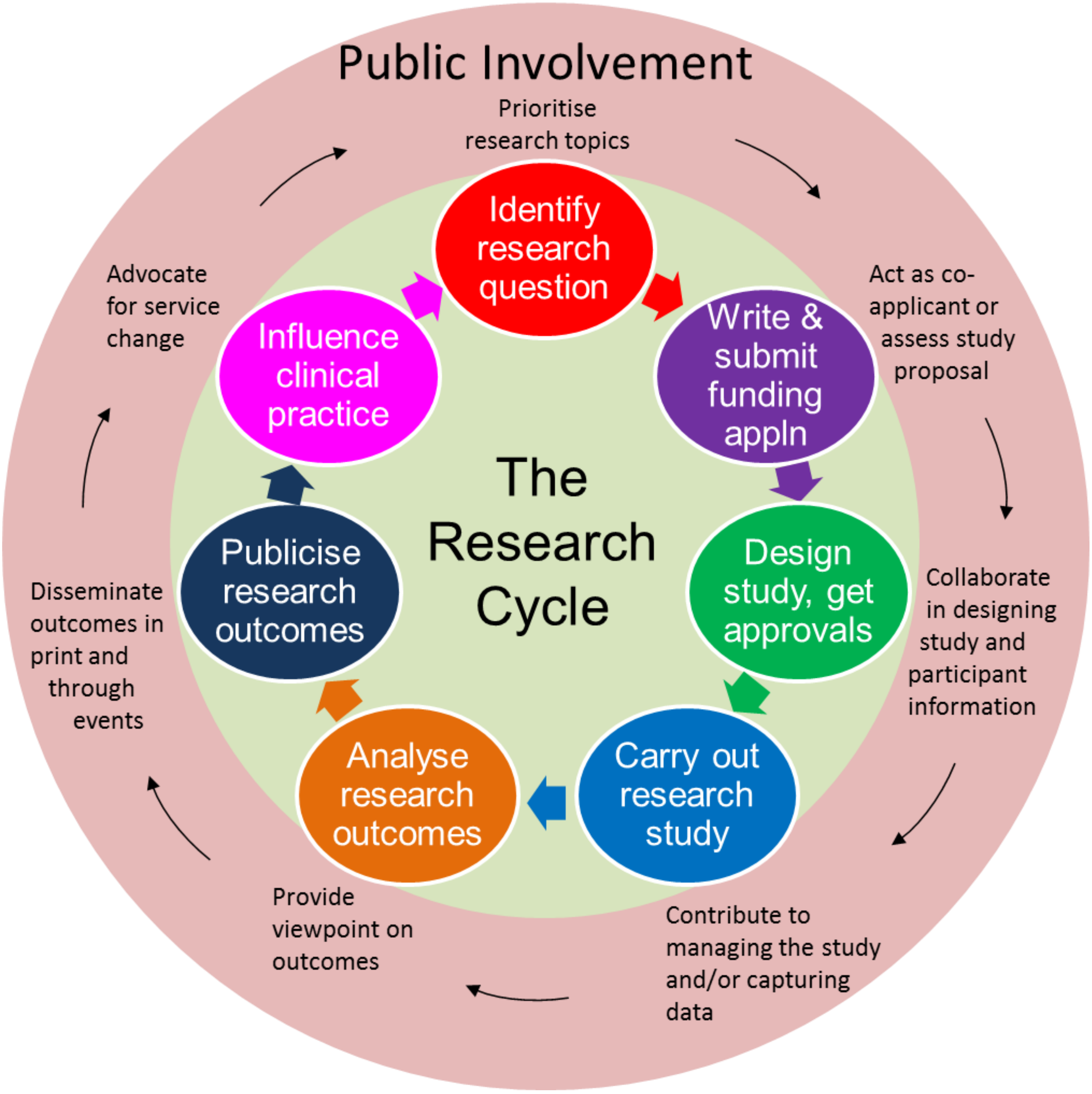

Guide co-produced by Dr Adele Horobin (NIHR Nottingham Hearing Biomedical Research Unit), Vikki Develin (Nottingham University Hospitals NHS Trust), Jane Flewitt (Research and Innovation NUH NHS Trust), Andy Wragg (NIHR Nottingham Digestive Diseases Biomedical Research Unit and Research and Innovation NUH NHS Trust), Veronica Colley (lay member), Fred Higton (lay member), Stevie Vanhegan (lay member), following consultation with members of the public attending lay assessor meetings in Nottingham, 2013.
