# Supplementary material for: Co-producing public involvement training with members of the public and research organisations in the East Midlands: creating, delivering and evaluating the lay assessor training programme
Source: Res Involv Engagem. 2017 Apr 5;3:7. doi: 10.1186/s40900-017-0056-0 (PMC5611661; doi:10.1186/s40900-017-0056-0)
Supplement: Supplementary file 1 — EV16012017 Dr Adele Horobin PPI Training programme evaluation Research Ethics Committee letter. (PDF 68 kb) [file 40900_2017_56_MOESM1_ESM.pdf]

E-mail: FMHS-ResearchEthics@nottingham.ac.uk

Faculty of Medicine and Health Sciences

Research Ethics Committee
C/o Faculty PVC Office
School of Medicine Education Centre
B Floor, Medical School
Queen's Medical Centre Campus
Nottingham University Hospitals
Nottingham
NG7 2UH

20th January 2016

Dr Adele Horobin
Patient and Public Involvement and Engagement Manager
National Institute for Health Research
Nottingham Hearing Biomedical Research Unit
Ropewalk House, 113 The Ropewalk
Nottingham
NG1 5DU

Dear Dr Horobin

Co-ordinating a regional approach to support for patient and public Involvement: the East Midlands training programme for lay assessors, Adele Horobin, George Brown, Fred Higton, Stevie Vanhegan, Andrew Wragg, Paula Wray, Dawn-Marie Walker.

Thank you for sending us the above paper that you have submitted for publication. I can confirm that this type of work and its methodology falls under the category of course evaluation, and would not have required full research ethics review or approval.

As somebody organising the course obtaining feedback from course participants about the course content, delivery and how it might be improved is considered part of routine activity expected in your daily professional role.

Yours sincerely

pp [illegible]

Professor Ravi Mahajan
Chair, Faculty of Medicine & Health Sciences Research Ethics Committee
